# Preliminary Analysis of Safety and Feasibility of a Single-Hole Laparoscopic Myomectomy via an Abdominal Scar Approach

**DOI:** 10.3389/fsurg.2022.916792

**Published:** 2022-07-11

**Authors:** Huimin Tang, Zhiyong Dong, Zhenyue Qin, Shoufeng Zhang, Huihui Wang, Weiwei Wei, Ruxia Shi, Jiming Chen, Bairong Xia

**Affiliations:** ^1^ Department of Gynecology, The Affiliated Changzhou No. 2 People’s Hospital of Nanjing Medical University, Changzhou, China; ^2^ Department of Gynecology, The First Affiliated Hospital of USTC, Division of Life Sciences and Medicine, University of Science and Technology of China, Hefei, China

**Keywords:** abdominal wall scar, single hole laparoscopic surgery, hysteromyoma, minimally invasive surgery, hidden scar

## Abstract

**Purpose:**

This paper aims to explore the safety and feasibility of a single-hole laparoscopic myomectomy through an abdominal scar approach.

**Method:**

The clinical data of seven patients who underwent the single-hole laparoscopic myomectomy *via* the abdominal scar approach from January to November 2021 in the Department of Gynecology, the Affiliated Changzhou No. 2 People's Hospital of Nanjing Medical University, were studied retrospectively. The duration of operation, the intraoperative blood loss, the decrease of postoperative hemoglobin, and the postoperative visual analogue score (0 points: no pain, 10 points: maximum pain) were recorded.

**Results:**

All seven patients received the operation successfully, without changing to the conventional laparoscopic operation or open appendectomy. The average blood loss was 101.42 ± 7.89 ml, the average length of hospital stay was 5 ± 0.53 days, the average operation duration was 130 ± 26.86 min, and the 24-h pain score was 1.57 ± 0.53. The seven patients had no intraoperative or postoperative complications and no damage to the ureter or bladder. All patients could urinate spontaneously without urinary retention or urinary tract infection after catheter removal. No analgesic drugs were used after the operation.

**Conclusion:**

The single-hole laparoscopic myomectomy *via* the abdominal scar approach is a more aesthetic and feasible option for eligible patients, but more cases and studies are needed for further confirmation.

## Introduction

Hysteromyoma is the most common gynecological benign tumor in women, especially women of childbearing age, accounting for about 20%–25% of women (1). Epidemiological statistics are far lower than the actual incidence. Although most patients have no clinical symptoms, 30% (2) of the patients still show symptoms such as increased menstruation, prolonged menstruation, anemia, frequent urination, urgent urination, and low back pain, which seriously affect the quality of life. As uterine leiomyoma is an estrogen-dependent disease, it often occurs in women of childbearing age. It is extremely rare in non-menarche women, and some fibroids may atrophy in peri-menopausal or postmenopausal women (2). For asymptomatic patients with uterine leiomyoma, regular follow-ups and treatments are often taken. For patients with symptoms, the current treatment primarily includes drug treatment, surgical treatment, and other interventional treatments. For symptomatic patients who have uterine leiomyoma but do not want to receive an operation, drugs can be used, such as progesterone, gonadotropin-releasing hormone agonist (GnRH-a), and mifepristone. The literature shows that oral progesterone can reduce symptoms or prevalence by 25% (3). GnRH-a treatment for 3 months can reduce the myoma and uterine volume by up to 50% (4), but the treatment is not well accepted because of the accompanying “quasi-menopause” symptoms. The reverse addition theory has been proposed to make up for this defect. Surgical treatment is feasible for uterine fibroids that lead to increased menstruation, anemia, frequent urination caused by bladder compression, and changes in defecation habits caused by rectal compression. For women with submucosal leiomyoma, the change in the endometrial environment affects fertility to a certain extent, increasing the rate of spontaneous abortion, and fertility can be improved after hysteroscopic treatment (3). For intramural myoma or subserous leiomyoma, transabdominal or laparoscopic surgery can be the option. Compared with laparotomy, laparoscopy has the advantages of less trauma, faster postoperative recovery, and less intraoperative bleeding (5). Since single-hole laparoscopy was first used for myomectomy (6), the operation has become increasingly mature after improvements. With people's aesthetic requirements increasing, single-hole laparoscopic myomectomy has been selected by more and more patients. Based on the successful fallopian tube recanalization by an abdominal scar approach (7), our hospital has combined the advantages of the two methods and completed the single-hole laparoscopic myomectomy through the abdominal scar approach, with achieving satisfactory results.

## Data and Methods

### General Data

Seven patients who underwent single-hole laparoscopic myomectomy *via* the abdominal scar approach from January to November 2021 were selected from Changzhou No. 2 People's Hospital Affiliated to Nanjing Medical University. The patients were 33–46 years old, with the average age being 38.71 ± 4.89 years, and the BMI was 22.52 ± 2.62 kg/m^2^ (Table 1). One of the patient cases was subserosal myoma, four cases were anterior intramural myoma, one case was multiple uterine myomas, and one case was broad ligament myoma. Among the seven patients, one had bilateral tubal ligation history and six had cesarean section history (two cases were a transverse scar of the cesarean section and four cases were a vertical scar of the cesarean section), including two with a vertical scar of the cesarean section combined with myomectomy history (Table 2).

(1) *Inclusion criteria:* (1) Previous history of abdominal incision; (2) indication of hysteromyoma surgery (4); and (3) patient's voluntary choice of the abdominal scar approach and signing the informed consent of the operation.
(2) *Exclusion criteria:* (1) Possibility of a malignant tumor; (2) body mass index (BMI) ≥ 30 kg/m^2^; and (3) patient with an underlying disease that is unsuitable for operation.

**Table 1:** Statistical data of the seven patients.

Characteristics	Mean ± standard deviation (*n* = 7)
Age (years)	38.71 ± 4.89
BMI (kg/m^2^)	22.52 ± 2.62
Intraoperative bleeding volume (ml)	101.42 ± 7.89
Operation duration (min)	130 ± 26.86
Postoperative hospital stay (days)	5 ± 0.53
Preoperative HB (g/L)	125.42 ± 14.63
Postoperative HB (g/L)	111.86 ± 16.10
VAS	1.57 ± 0.53

*BMI, body mass index; HB, hemoglobin; VAS, visual analogue score.*

**Table 2:** General information of patients.

Case	Age (years)	BMI (kg/m^2^)	Reproductive history	Surgical history	Scar site of the abdominal wall
1	37	26.44	G5P2	History of cesarean section, history of myomectomy	Vertical scar of the abdominal wall during cesarean section
2	34	26.51	G5P3	History of cesarean section	Vertical scar of the abdominal wall during cesarean section
3	37	20.88	G5P1	History of cesarean section	Vertical scar of the abdominal wall during cesarean section
4	46	20.81	G1P1	History of cesarean section	Transverse scar of the abdominal wall during cesarean section
5	46	22.67	G3P1	History of tubal ligation	Abdominal tubal ligation scar
6	33	20.32	G3P1	History of cesarean section	Transverse scar of the abdominal wall during cesarean section
7	38	20	G2P1	History of cesarean section, history of myomectomy	Vertical scar of the abdominal wall during cesarean section

*Reproductive history: G, gestation; P, production.*

### Operation Method

#### Preoperative Preparation

All seven patients underwent general anesthesia; fasting and water deprivation 10 h before the operation to prepare for anesthesia; skin preparation and vaginal cleaning; diet preparation 3 days before the operation to improve the intestinal environment and reduce the impact of the intestinal tract on operation; disinfection of the scar to reduce postoperative infection; and preoperative education by nurses to patients before the operation to reduce their tension.

#### Equipment Preparation

A complete set of digital systems for laparoscopy (such as lens, display, pneumoperitoneum system, light source system, and recorder), instrument set for conventional gynecological transabdominal surgery, and the items required for single-hole laparoscopy (such as the port and protective ring required for access, as well as the lens, operating instruments, and suture required for laparoscopy) were kept ready.

#### Surgical Procedures

**Figure 1:**
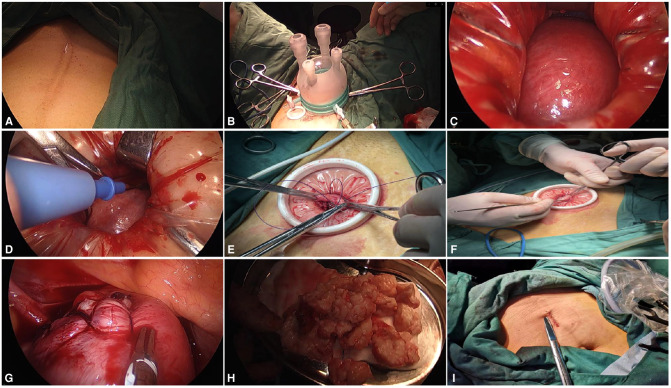
(**A**) Incision through abdominal wall scar; (**B**) connecting the single-hole port; (**C**) using the uterine lifter to push the uterus to the incision; (**D**) dissecting the tumor with an electric knife under direct vision; (**E**) suturing the uterus under direct vision; (**F**) removing the tumor from the incision in blocks; (**G**) laparoscopic exploration and hemostasis; (**H**) stripped tumor; and (**I**) suturing abdominal incision.

The patient took the bladder lithotomy position (kept the head low and foot height ≥30° and the abduction of both legs <90°). After the general anesthesia was satisfactory, the patient received routine disinfection and was draped, and the assistant placed the uterine lifting device and retained the catheterization. The transabdominal scar approach was adopted. Taking the scar after a cesarean section as an example, first, an incision of 2.0 cm was made in the lower part of the scar of the original cesarean section (Figure 1A) layer by layer into the abdomen. Then, the incision protection ring and the single-hole laparoscopic special port was connected (Figure 1B), the disposable single-hole flexible sheath was fixed, the CO_2_ gas was filled until the abdominal pressure reached 14 mmHg, and then the 30° laparoscopic lens and other operating instruments were placed. Single-hole laparoscopy was used to detect the abdominal adhesion and separate the adhesion with an ultrasound knife to restore the normal pelvic structure. The laparoscopic device was removed, the abdominal wall was gently lifted with a thyroid retractor, and the uterus was pushed to the abdominal wall incision by using the uterine device in conjunction (Figure 1C). The inject diluted vasopressin into the myometrium locally (avoiding the tumor). Under direct vision, the serous layer was cut open on the surface of the tumor with an electric knife, and the tumor was stripped bluntly and sharply with fingers or the electric knife (Figure 1D). The operator can extend his/her finger from the incision into the pelvic cavity and cooperate with the uterine lifter to carefully check for any other suspicious tumor tissue (for uterine leiomyoma in the posterior wall, the approach can be on the upper part of the scar and laparoscopy can be used to peel off the tumor as much as possible). Under direct vision, the myometrium and serosa of the uterine wound were sutured, the wound was closed, the dead space was avoided, and the uterine body was formed (if a certain wound surface was located in the posterior wall or it was difficult to be sutured directly, the laparoscopic device can be connected for fine suture) (Figure 1E). Under direct vision, the tumor was cut, removed with a scalpel or scissors, and sent for pathological examination as necessary (Figure 1F). The single-hole laparoscopic device was connected, the pelvis was rinsed with normal saline to avoid residue, any active bleeding of the wound was checked under the microscope (Figure 1G), the instrument was removed to empty the gas, and the abdominal cavity was closed layer by layer.

#### Postoperative Treatment

All seven patients returned to the ward smoothly without intraoperative complications. Oxytocin was given to facilitate uterine contraction and rehydration, and antibiotics were given to prevent infection as necessary. The dressing was changed after the operation.

#### Observation Indicators

The operation duration, intraoperative bleeding, intraoperative/postoperative complications, preoperative and postoperative hemoglobin, postoperative hospital stay, postoperative visual analogue scale (pain score scale: 0–10 represents the degree of pain from painless to intolerable pain), and grade of incision healing (Grade A refers to one-time wound healing without infection after stitch removal, Grade B refers to incision infection and healing after treatment, and Grade C refers to incision rupture or infection without healing) were used.

### Postoperative Follow-Ups

Six months after the operation, follow-ups were conducted to check for any long-term complications such as incision hernia and myoma with recurrence in a short time. The follow-up results showed that the seven patients had no incision hernia and no abnormality was found in vaginal ultrasound. The symptoms of patients were significantly improved and followed up on regularly.

### Statistical Analysis

SPSS Statistics was used for data statistics, and the data meeting the normal distribution conditions were expressed as mean ± standard deviation (*x* ± *s*).

## Results

All seven patients received the operation successfully, without changing to the conventional laparoscopic operation or gynecological transabdominal surgery. The ureter or bladder was not injured during the operation, and the urine was clear after the operation. The postoperative hospital stay was 4–6 days, with an average of 5 ± 0.53 days. The average postoperative blood loss was 101.42 ± 7.89 ml, and the postoperative visual analog score was 1.57 ± 0.53. All patients had exhausted, catheter removal was performed 1–2 days after the operation, and the postoperative incision healed well, without complications such as wound infection and bleeding (Table 3). Abdominal drainage tubes were placed in two patients and removed on the second day. The incision sites of the seven patients were original abdominal wall scars and were not changed to traditional laparoscopy or laparotomy.

**Table 3:** Single-hole laparoscopic myomectomy *via* the abdominal scar approach.

Case	Number of myomas (piece)	Myoma size (cm)	Myoma location	Operation duration (min)	Intraoperative bleeding volume (ml)	Preoperative hemoglobin (g/L)	Postoperative hemoglobin (g/L)	Pathology	Grade of incision healing	Postoperative visual analogue score
1	1	6.2×4.5×5.9	Left broad ligament	125	105	139	117	Cellular leiomyoma with steatosis	A	Two
2	1	7.3×6.0×5.3	Anterior wall	120	105	127	118	Leiomyoma	A	One
3	2	Front wall 1 piece: 6.5×6.0×6.0; Rear wall 1 piece: 1.8×1.0×1.0	Front wall 1 piece and Rear wall 1 piece	110	110	118	124	Leiomyoma	A	Two
4	10	Maximum myoma: 8.1×5.1×7.9	Intramural (maximum in posterior wall)	180	90	96	79	Leiomyoma with degeneration	A	Two
5	1	7.0*6.0×5.0	Posterior wall	160	110	128	108	Leiomyoma	A	One
6	1	4.5×5.0×5.0	Anterior wall	115	90	145	133	Leiomyoma	A	One
7	2	Front wall 2 pieces: 6.0×5.0×5.0 and 4.0×4.5×4.5	Anterior wall	100	100	125	104	Leiomyoma	A	Two

## Discussion

Hysteromyoma is the most common gynecological benign tumor for women of childbearing age, and its etiology is not clear, which may be related to the patient's age, not having a child or late childbearing, obesity, and other factors (4). Most patients have no clinical symptoms, and only a small number of patients have symptoms such as increased menstruation, increased vaginal secretions, and abdominal pain. For women who have fertility requirements or want to retain the uterus, hysteromyoma removal is a relatively safe and feasible method. Compared with open surgery, traditional laparoscopy or single-hole laparoscopy has a better cosmetic effect, faster postoperative recovery, and shorter hospital stay (8). However, traditional laparoscopy needs to insert 3–4 puncture devices, which increases the incidence of abdominal incision hernia to a certain extent, and the injury rate of intestinal tubes and blood vessels during puncture also increases (9). With people's aesthetic requirements increases, LESS came into being. Wheelless (10) first applied single-hole laparoscopy to gynecological tubal ligation as early as 1969. However, due to the different operation modes of single-hole laparoscopy, the operation is more difficult, which often leads to a longer operation time than traditional laparoscopy. Besides, after transumbilical single-hole laparoscopy, the umbilical hole is more difficult and the umbilical hole plastic surgery is also challenging. Many beginners have encountered the conditions of red and swollen umbilical hole tissue, seepage, necrosis, and infection after suture, resulting in the limited application of single-hole laparoscopy (11). The transabdominal scar approach can avoid umbilical hole plastic surgery and the resulting risks to a certain extent. Meanwhile, the transabdominal scar approach can reduce the formation of new abdominal scars and hide new surgical scars with the original scars to form “hidden scars” (12).

Taking the cesarean scar on the abdominal wall as an example, its location is closer to the pelvic cavity and the uterus than the single-hole laparoscopy through the umbilical approach. It has the following advantages and disadvantages: (1) The operator can cooperate using a 30° laparoscopic lens, which has a wider field of vision and more convenient operation. The surgical instruments that are double curved or of different lengths can be used to avoid the “chopstick effect” of laparoscopic surgery (13), which also helps shorten the operation duration. After stripping the tumor body in the field of vision, the operator can penetrate the finger into the pelvic cavity through the incision channel and scatter the small tumor body through tactile perception, the mode that helps accurately identify the scattered small tumor body of the uterus, to peel off the myoma as much as possible and reduce the recurrence of postoperative hysteromyoma and damage to the myometrium caused by blind exploration of myoma by instruments. The authors’ team has also used the “finger probe method” to complete the traditional laparoscopic myomectomy (14), which is the advantage of this method. (2) For patients with hysteromyoma on the anterior wall, they can choose to cut near the scar of the uterine fundus, combine the transabdominal operation with the pneumoperitoneum-free single-hole laparoscopic operation, cooperate with the uterine lifting device, and use the laparoscopic surgical instruments under direct vision. This method can increase the surgical field, reduce intraoperative bleeding, facilitate the suture procedure of the laparoscopic operation, and simplify the operation to a certain extent. Meanwhile, the thyroid retractor is used to gently lift the skin to avoid the damage of gram steel needle to the abdominal wall. For patients with uterine fibroids in the anterior wall, this is a more feasible scheme with a higher aesthetic value. The authors' team also completed several cases of pneumoperitoneum-free single-hole laparoscopic ovarian cyst stripping (15). For patients with posterior wall hysteromyoma, the upper abdominal scar approach can be selected, combined with laparoscopic assistance, which can address the disadvantage of small pelvic operation space through the abdominal scar approach and improve the safety of the operation. (3) The scar of the cesarean section is closer to the uterus, the operation space of laparoscopic instruments is small, and the operation is difficult. In comparison, the transumbilical approach is easier to lead to the “conflict” of surgical instruments, and the “chopstick effect” is more serious. Therefore, the operator is required to have better operation skills. Especially for patients with the transverse scar of cesarean section, because their transverse scar is close to the bladder, the operation may easily damage the bladder, and the operator is required to pay more attention to the anatomical level when entering the abdomen layer by layer. Patients should be evaluated before the operation, and other operation methods should be chosen for patients not suitable for this operation. (4) Compared with laparoscopy-assisted mini-laparotomy, the transabdominal scar approach can reduce the number of abdominal wall puncture holes. This operation is based on the abdominal wall scar; the incision is smaller, without creating a new abdominal wall scar and is more aesthetically pleasing. Meanwhile, it can fine-suture the uterus and reduce the bleeding of uterine wounds. If the position of uterine leiomyoma is difficult to suture directly, the laparoscopy can be used without adding new puncture holes. (5) For patients with a history of abdominal wall scar, the most noticeable problem is abdominal adhesion. According to research, the risk of postoperative adhesion can reach 90% regardless of the operation method adopted (16, 17), which greatly increases the difficulty of laparoscopic operation, as well as the probability of damaging pelvic and abdominal organs and the incidence of a change to transabdominal operation (18). For abdominal organ adhesion, due to the lower local heat generated by the ultrasonic scalpel and less thermal damage to the tissue, the ultrasonic scalpel is the best choice for separating adhesion (19). Because the patient has a history of surgery, the intestinal canal may adhere to the original surgical incision, which requires the operator to pay attention to the layers when entering the abdomen and be more cautious in the operation. After the incision protective ring is placed, the laparoscopic lens can be used to detect the adhesion of the abdominal wall and intestinal canal, and the patient should be changed to transabdominal operation as necessary to ensure safety.

In general, the single-hole laparoscopic myomectomy *via* abdominal scar approach is more in line with the aesthetic requirements of patients. It is a safe and feasible scheme for patients who meet the inclusion criteria of surgery. However, if it is widely used, more randomized controlled studies are needed to further validate its effectiveness and feasibility.

## Data Availability

The raw data supporting the conclusions of this article will be made available by the authors, without undue reservation.
